# Insomnia in teachers with the resumption of in-person instruction at schools amidst the COVID-19 pandemic: A cross-sectional study

**DOI:** 10.12688/f1000research.141274.1

**Published:** 2023-09-26

**Authors:** Sowmini Padmanabh Kamath, Prasanna Mithra, Padmanabh Kamath, Bhaskaran Unnikrishnan

**Affiliations:** 1 Department of Pediatrics, Kasturba Medical College, Mangalore, Manipal Academy of Higher Education, Manipal, India; 2 Department of Community Medicine, Kasturba Medical College, Mangalore, Manipal Academy of Higher Education, Manipal, India; 3 Department of Cardiology, Kasturba Medical College, Mangalore, Manipal Academy of Higher Education, Manipal, India

**Keywords:** Anxiety, COVID-19, Cross sectional studies, Humans, Pandemics, Depression, Insomnia, Schools

## Abstract

Background: The coronavirus (COVID-19) pandemic has affected people's economies, lifestyles, and physical, emotional, and sleep health. This research aimed to estimate the prevalence of insomnia and symptoms of stress, anxiety, and depression among teachers with the resumption of in-person instruction at schools following a hiatus after COVID-19 lockdowns in India. We also studied the association of teachers' insomnia with psychological symptoms and demographic variables.

Methods: We conducted a cross-sectional survey between October –November 2021 after schools had reopened during the COVID-19 pandemic. Data was collected using standard questionnaires online among schoolteachers. We explored the association of insomnia with teachers' symptoms of stress, anxiety, depression, sex, school boards, and age groups.

Results: Of 124 schoolteachers surveyed, the prevalence of insomnia was 37.9% (subthreshold in 25% and clinical in 12.9%). The prevalence of stress, depression, and anxiety was 20.2%, 30.6%, and 45.2%, respectively. There was a significant association (p<0.001) of insomnia with symptoms of anxiety, stress, and depression in univariate analysis. On multivariate analysis, we found that those feeling stressed had a 6.4 times higher risk of insomnia (95% CI: 1.5-28.3, p - 0.01). There was no association of insomnia with age, sex, school educational boards, and type of institution.

Conclusions: Over one-third (37.9%) of teachers reported having trouble sleeping when they returned to the school's typical face-to-face instruction modalities through COVID-19 times, and insomnia was more prevalent in those with stress.

## Introduction

Insomnia is the most frequent sleep disorder encountered among the public. Disturbances in sleep following significant stressful situations, including natural disasters, have been reported previously. ^1^ The COVID-19 pandemic has caused crises globally, causing significant changes in the lifestyle of people.

The pandemic has resulted in anxiety, stress, worries about one's health and family, job insecurities, economic instabilities, financial crises, and challenges in managing work and family duties. In addition, people had to follow COVID-appropriate behavior resulting in lesser social contacts and interactions. Uncertainties and increasing unprecedented changes occurred worldwide, and people began encountering impairment in sleep and altered circadian rhythms. Hurley refers to the sleep disturbances related to the pandemic as “coronasomnia or covidsomnia”. ^2^

The teaching profession is stressful, and teachers have reported symptoms of stress, ^3^^,^^4^ anxiety, and depression ^5^ along with sleep problems ^6^ even during pre-covid times. With the onset of the COVID-19 pandemic, there was a significant shift from conventional pedagogy teaching to e-learning methodology. Teachers had to adapt to the online teaching mode abruptly and balance their duties to their families during the pandemic crises. Most teachers were unfamiliar with e-learning and worried about delivering quality teaching, especially for children from lower socioeconomic status and remote villages who still needed internet access. ^7^^–^^10^

The effect of the COVID-19 pandemic on psychological symptoms in various populations, ^11^^,^^12^ including teachers, has been studied previously, ^13^^,^^14^ along with disturbances in sleep in the general public have been documented during the COVID-19 lockdowns. ^15^^,^^16^ Adapting to e-learning increased stress for the schoolteachers and affected their sleep. ^17^ More than half of the teachers in the USA during COVID-19 have reported insomnia. ^18^

A survey in the United Kingdom during the COVID-19 pandemic revealed an increase in sleeplessness from pre-pandemic values of 15.7% to 24.7%. ^19^ Another study showed changes in sleep schedules and quality/quantity of sleep at nighttime. ^20^ There is a link between insomnia severity with signs of despair, anxiety, and poor sleep hygiene. ^21^ Female sex is usually predisposed to insomnia. ^16^^,^^19^ On the contrary, an Indian survey among the public did not show statistically significant differences with sex. ^15^ Further, factors such as having young children, perceived financial difficulties, and the presence of symptoms of COVID-19 were predictive of sleep loss in yet another study. ^19^

The long-term effects of insomnia include mood disorders, mental changes, poor cognitive performances, diabetes, hypertension, overweight/obesity, cerebrovascular, cardiovascular diseases, and metabolic syndrome, with an increase in mortality. ^22^ Thus it is essential to recognize and address sleep issues by coping mechanisms to improve sleep hygiene.

As the pandemic continued, The Ministry of Education, India, ^23^ initiated the reopening of schools and educational institutions from the online teaching mode to in-person instructive classes in a gradational manner following the second COVID-19 wave in India. There was fear of a resurgence of infection and additional responsibility to maintain implemented COVID-appropriate behaviors at schools and manage the children once schools reopened. So reverting to regular face-to-face teaching in schools would be challenging for the teachers and students, especially when there was an impending third wave.

Following a hiatus of school closure during the pandemic, in the context of face-to-face teaching mode when schools reopened, the teachers' psychological symptoms were high in a Spanish study. ^24^ Similarly, our study reported a high prevalence of psychological symptoms among schoolteachers after schools reopened for regular face-to-face instruction. ^25^ The data for the current research was also gathered concurrently from the same group of teachers as part of a more extensive study. We used the English version of the Insomnia Severity Index (ISI) questionnaire ^26^ to assess insomnia levels. The ISI questionnaire is valid and reliable for usage in the Indian population. ^27^

Despite the volume of evidence on psychological symptoms and insomnia in various populations, ^28^^,^^29^ limited literature is available on teachers' insomnia levels during the pandemic. Additionally, this research focused on instructors and looked at a specific time during the COVID-19 outbreak when Indian schools were moving from a complete lockdown to a gradual resumption of in-person activities.

Therefore, this study's primary goal is to assess teacher's insomnia and its association with their psychological states and demographic variables at a crucial juncture during the COVID-19 crisis, especially in the scenario when schools and educational institutes were reopening after a hiatus to contain the spread of COVID-19 from March 2020 to October 2021.

## Methods

### Study design and participants

**Figure 1.**
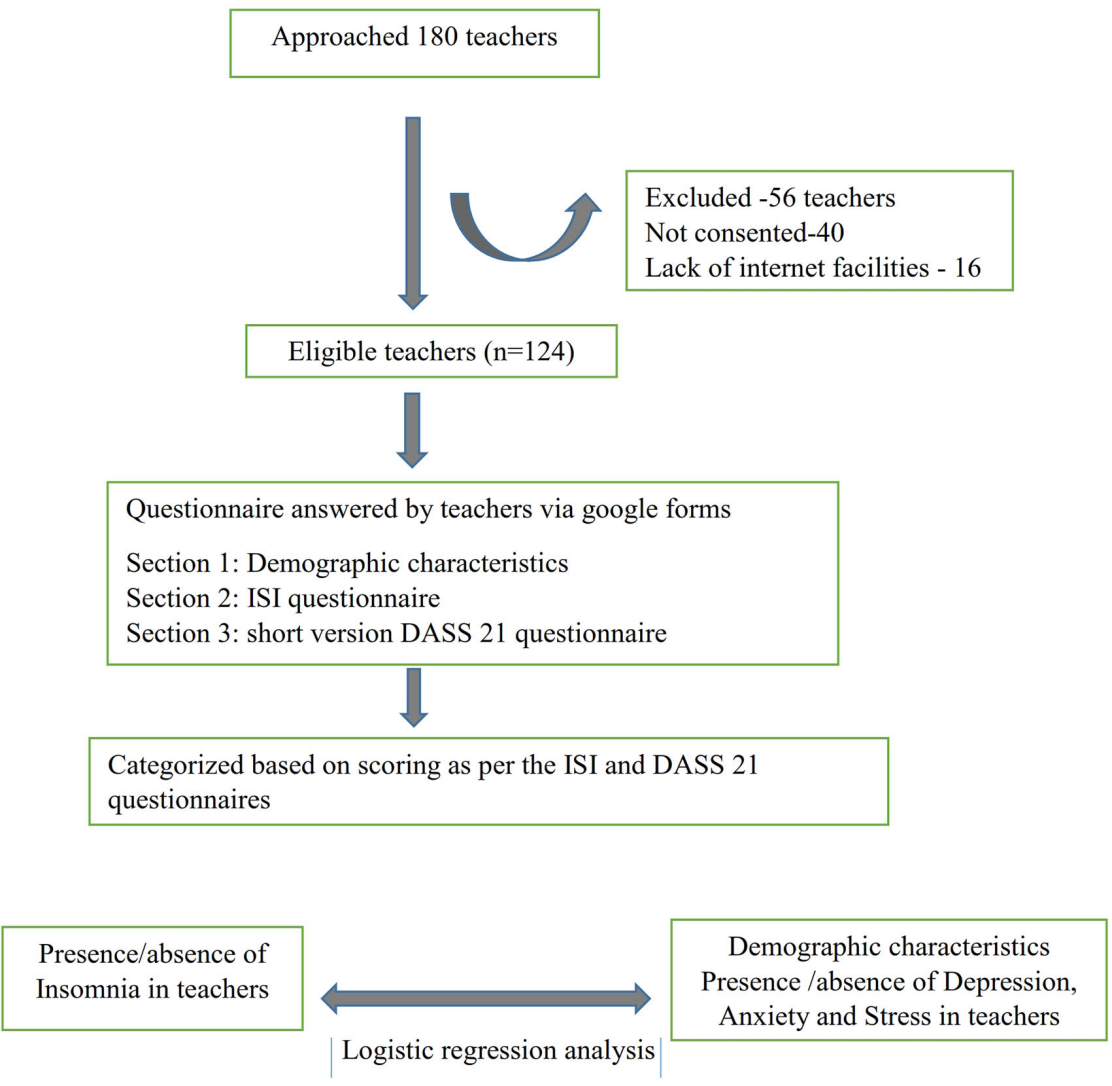
Study flow diagram.

We conducted a cross-sectional study involving teachers in a few chosen schools in the Dakshina Kannada district, situated in a state in southern India. The study duration was two months (November to December 2021). All teachers who agreed to participate in the study were incorporated. We excluded teachers with no Internet access and those who could not enroll owing to ongoing medical conditions. The STROBE (Strengthening the Reporting of Observational Studies in Epidemiology) statement ^30^ is adhered to in this study and a completed checklist is provided in the *Reporting guidelines.* ^31^ Figure 1 depicts the study flow as per the STROBE criteria.

### Sample size

The sample size calculation is based on teachers' anxiety levels estimated to be 49.4 %, ^24^ similar to insomnia prevalence of 52 % in another study. ^18^ Considering an 80% power, a 95% confidence level, a 10% relative precision, and a 20% non-response rate, we determined the sample size to be 115. Our study on the teachers levels of depression anxiety and stress was reported earlier ^25^ ( https://doi.org/10.12688/f1000research.110720.2) with the same data collection procedures as the current study.

### Ethical considerations

The conduct of the study adhered to the 1964 Declaration of Helsinki and its later amendments, as well as other relevant ethical standards. The institutional ethics committee of Kasturba Medical College, Mangalore, Manipal Academy Of Higher Education, Manipal, India authorized the study (approval number: IEC KMC MLR -06/2020/184). We took permission from the Block Education Officer (BEO). Further, we obtained permission from the school administration in charge and the principals of the selected schools. The participant information sheet (as in *Extended Data*) ^31^ provided the purpose of the study. We collected informed consent (as in *Extended Data*) ^31^ and permission to publish from the teachers.

### Data collection

The Block Education Officer's (BEO) office furnished us with a school list of our district. There were an equal number of private and public schools in the sample. We listed the schools in increasing order of teacher count. Then, we chose schools by lottery until we obtained the necessary sample size. The BEO and the concerned school administration consented once we outlined the specifics of the study. We prepared the questionnaire using the online data collection tool Google Forms. Through WhatsApp and school email, we shared the questionnaire link, the participant information sheet having study details, the Block Education Officers letter of authorization, and the IEC certificate with the head teacher or principal of the selected school. At the investigator's request, the principal sent the link to each school's WhatsApp group for teachers to answer the questionnaire link. We gathered informed consent through the questionnaire link. After responding to the question in hand, teachers had to scroll down to the following ones because it was necessary to answer all the questions in the link. The participants could respond to the questionnaires in less than ten minutes.

### Tools

We gathered data through a questionnaire using Google Forms. The questionnaire (provided as *Extended Data*) ^31^ consisted of Section 1 for demographic details; Section 2 comprised the insomnia severity index (ISI) scales for insomnia; and Section 3-featured Depression, anxiety, and stress scales (DASS-21) to measure stress and anxiety and depression levels. Two weeks after returning to their jobs at the school, we ensured the questionnaires were accessible to the teachers.

A) ***Insomnia Severity Index (ISI)***

The Insomnia severity index (ISI) is a seven-item screening tool for insomnia. ^26^^,^^32^ The participants need to rate their sleep-related issues using a Likert scale. Questions pertain to subjective sleep quality, symptom severity, sleep pattern satisfaction levels, day-to-day functioning by insomnia, others' observation of the participant's insomnia, and the overall distress because of insomnia. The questionnaire evaluates the severity of recent two-week bouts of insomnia. The item responses (ranging from 0-4) with higher scores indicated severe/acute insomnia symptoms. The seven items' scores were added (total score ranges from 0-28), and we classified insomnia into the following four groups:

Moderately severe clinical insomnia (score of 15-21).

Severe clinical insomnia (score of 22–28).

Subthreshold insomnia (score of 8-14), and

No clinically relevant insomnia (scores of 0-7)

We procured permission to use the ISI questionnaire (English version) from the author through Mapi Research Trust ( https://eprovide.mapi-trust.org/instruments/insomnia-severity-index).

B) ***Depression, anxiety, and stress (DASS 21) scale***

The English short version of the DASS 21 questionnaire was used ( https://eprovide.mapi-trust.org/instruments/depression-anxiety-stress-scales). The self-report questionnaire is designed to identify the psychological conditions of stress, depression, and anxiety. With seven items in each domain, the twenty-one statements are organized for three scales: depression, anxiety, and stress. Each statement on the scale has four alternative answers (scores 0- does not apply to me; 1-applied to me to occasionally or to some extent; 2-applied to me frequently or significantly; and 3-applied to me frequently or a lot). The replies show how much each respondent found the statement true over the previous week. The scores for relevant items from each domain were pooled and multiplied by two to determine the final scores. We categorize the severity of the symptoms into five groups based on the cut-off scores as no symptoms, minor symptoms, moderate symptoms, severe symptoms, and extremely severe symptoms, which are represented by the numbers I through V. ^33^

Additionally, we divided the symptoms into categories based on their presence or absence, such as “insomnia absent and insomnia present,” “depression absent and depression present,” “anxiety absent and anxiety present,” and “stress absent and stress present.” We compared the presence/absence of insomnia with sex, age groups, school boards, type of school, and presence/absence of psychological symptoms.

### Study groups and definitions

Schools run or assisted by the government are categorized as public schools. The Council for the Indian Secondary Certificate Examination (CISCE), a private board of school education in India, administers the Indian Certificate of Secondary Education (ICSE). The Government of India supervises the Central Board of Secondary Education (CBSE), a national-level board of education in India for both public and private schools. Karnataka State Board manages the State Education Examination Board. To compare the levels of stress, depression, and anxiety among teachers, we divided school education boards into (CBSE+ICSE) against state boards and school institution types into public versus private schools.

### Data analysis

IBM SPSS Statistics for Windows, Version 25.0. Armonk, NY: IBM Corp. analyzed the information gathered. We used the appropriate tables and figures to express the results as proportions. To compare the groups, we used Chi-square tests. We used Logistic regression analysis to calculate the odds ratio of the variables contributing to teachers' insomnia risk. A p-value of less than 0.05 was used to denote statistical significance.

## Results

Of 180 teachers contacted, 124(68.89%) ^31^ completed the survey. Of 124 teachers, 108(87.1%) were females, and nearly 30% of teachers were aged less than 40 years. About 112 (90.3 %) were teaching at private institutions, while 70 (56.5%) and 54 (43.5%) were teaching in the central boards (CBSE+ICSE) and state boards of education, respectively ( Table 1). The full raw data can be found under *Underlying data.* ^31^

**Table 1.** Basic demographic characteristics of school teachers.

Variables	N (%)
**Age groups**	
<40 years	37 (29.8)
≥40 years	87 (70.2)
**Sex**	
Female	108 (87.1)
Male	16 (12.9)
**School board**	
State board	54 (43.5)
CBSE+ICSE	70 (56.5)
**Institution**	
Government aided institutes	12 (9.7)
Private institution	112 (90.3)

CBSE: Central Board of Secondary Education; ICSE: Indian Certificate of Secondary Education.

The prevalence of insomnia was 37.9% (subthreshold in 25% and clinical in 12.9%). The prevalence of stress, depression, and anxiety was 20.2%, 30.6%, and 45.2%, respectively ( Table 2).

**Table 2.** Prevalence’s of insomnia and psychological symptoms (depression, anxiety and stress) in school teachers.

Symptomatology	Presence of symptoms N (%)	Absence of symptoms N (%)
Insomnia	47 (37.90)	77 (62.10)
Depression	38 (30.6)	86 (69.4)
Anxiety	56 (45.2)	68 (54.8)
Stress	25 (20.2)	99 (79.8)

The frequency of teacher’s responses towards the ISI questionnaires is depicted in Table 3. Depressive symptoms were present in 38 (30.6%), with severity varying from mild (12.9%) to moderate (9.7%) to severe (5.6%) to extremely severe (2.4%). Anxiety was expressed by 56(45.2%), with severity being mild in 17.7%, moderate in 16.9%, severe in 3.2%, and extremely severe in 7.3%. Stress symptoms were seen in 25(20.2%), with variations being mild (12.1%), moderate (3.2%), severe (3.2%), and extremely severe (1.6%).

**Table 3.** Frequency of the teacher’s responses towards the ISI questionnaire.

Sl no	Variables	n (%)
1a.	Difficulty falling asleep	
	None	57 (46)
	Mild	35 (28.2)
	Moderate	27 (21.8)
	Severe	4 (3.2)
	Very severe	1 (0.8)
1b.	Difficulty staying asleep	
	None	66 (53.2)
	Mild	33 (26.6)
	Moderate	21 (16.9)
	Severe	3 (2.4)
	Very severe	1 (0.8)
1c.	Problems waking up too early	
	None	47 (37.9)
	Mild	45 (36.3)
	Moderate	23 (18.5)
	Severe	7 (5.6)
	Very severe	2 (1.6)
2	How SATISFIED/DISSATISFIED are you with your CURRENT sleep pattern?	
	Very satisfied	24 (19.4)
	Satisfied	49 (39.5)
	Neutral	34 (27.4)
	Dissatisfied	17 (13.7)
	Very dissatisfied	-
3	To what extent do you consider your sleep problem to INTERFERE with your daily functioning (e.g. daytime fatigue, mood, ability to function at work/daily chores, concentration, memory, mood, etc.) CURRENTLY?	
	Not at all interfering	53 (42.7)
	A little	44 (35.5)
	Somewhat	16 (12.9)
	Much	10 (8.1)
	Very much interfering	1 (0.8)
4.	How NOTICEABLE to others do you think your sleep problem is in terms of impairing the quality of your life?	
	Not at all noticeable	61 (49.2)
	A little	43 (34.7)
	Somewhat	15 (12.1)
	Much	3 (2.4)
	Very much noticeable	2 (1.6)
5.	How WORRIED/DISTRESSED are you about your current sleep problem?	
	Not at all	59 (47.6)
	A little	46 (37.1)
	Somewhat	9 (7.3)
	Much	8 (6.5)
	Very much	2 (1.6)

On univariate analysis, stress, anxiety, and depression were significantly associated (p<0.001) with insomnia in teachers. On multivariate analysis ( Table 4), only stress in teachers was an independent factor for insomnia (95% CI: 1.5-28.3, p -0.01). Those teachers who were stressed out had a 6.4 times higher incidence of insomnia (odds ratio). The presence of anxiety or depressive symptoms, sex, school educational boards, or the type of institution did not significantly correlate with insomnia in teachers.

**Table 4.** Logistics regression showing the association of demographic and psychological factors with insomnia among school teachers.

Characteristics		Insomnia (n=47) n (%)	Unadjusted OR (95% CI)	Adjusted OR (95% CI)	p value
**Age groups (years)**	<40 (n=37)	18 (48.7)	1.9 (0.87-4.1)	2.5 (0.9-6.3)	0.05
	≥40 (n=87)	29 (33.3)	1	1	
**Sex**	Male (n=16)	9 (56.3)	2.4 (0.8-6.9)	1.67 (0.46-6.1)	0.44
	Female (n=108)	38 (35.2)	1	1	
**Depression**	Present (n=38)	27 (71.1)	8.1 (3.4-19.2)	2.6 (0.79-8.60)	0.12
	Absent (n=86)	20 (23.3)	1	1	
**Anxiety**	Present (n=56)	33 (58.9)	5.5 (2.5-12.2)	1.5 (0.54-4.4)	0.42
	Absent (n=68)	14 (20.6)	1	1	
**Stress**	Present (n=25)	21 (84.0)	14.7 (4.6-47.0)	6.4 (1.5-28.3)	**0.01** *
	Absent ( n=99)	26 (26.3)	1	1	

* OR-Odds ratio from binary logistic regression, p value of <0.05 was considered as statistically significant.

## Discussion

We observed that 37.9 % of teachers expressed the presence of insomnia (subthreshold - 25 % and clinical -12.9 %) after resuming face-to-face classes at school amid the COVID-19 pandemic. Similarly, a review study documented that the COVID-19 pandemic is linked with a significant increase in subthreshold insomnia but not with moderate or severe symptoms of insomnia. ^34^

As per the United Kingdom's “Education Support” charity's annual teacher well-being survey findings, 52% of teachers have experienced trouble sleeping during the pandemic, up from 37% who claimed they battled with sleep in the preceding two years. ^18^ The increased prevalence in the United Kingdom was most likely because the study data were collected during the peak of the COVID-19 pandemic when people were alarmed about the COVID-19 crisis, had economic concerns, and had societal constraints.

A study showed that teachers with distressed personalities suffered more often from significant insomnia and depression. ^35^ A known risk factor for the emergence of depression is insomnia. Depression is known to increase a person's negative attitude about their work and responsibilities, which in turn negatively affects the manner and effectiveness of their work.

During the pandemic, moderate and severe clinical insomnia was present in 13.32 % and 1.85% of the Indian adult population, respectively. ^15^ The general population in another Indian study reported poor sleep quality in higher numbers (57.2%), with quality of sleep significantly associated with self-reported mental health status. ^36^ Across various populations, the prevalence of insomnia was 34 to 36 % in medical staff, ^37^ 20.5% in public China, ^16^ 31.3%, ^38^ and 43.6 % ^39^ in university students and 23.2 % ^40^ in adolescents and young adults in China.

As per a systematic review and meta-analysis, the corrected pooled estimated prevalence of sleep difficulties among health care professionals (HCP), the general population, and COVID-19 patients was 31%, 18%, and 57%, respectively. ^41^ According to another meta-analysis, the HCP and general population had similar sleep issues of 36.0% and 32.3%, respectively, and with a higher pooled prevalence of 74.8% among COVID-19 patients. ^42^

We found that 20.2%, 30.6%, and 45.2% of teachers had expressed psychological symptoms of stress, depression, and anxiety, respectively. There was a higher prevalence of stress (50.6%), depression (32.2%), and anxiety (49.4%) among teachers who had face-to-face instruction with the reopening of schools in Spain. ^24^ Symptoms of stress, depression, and anxiety were present in teachers at rates of 30 %, 19%, and 17%, respectively, in a systematic review and meta-analysis. ^13^ A similar systematic analysis among teachers found anxiety in 10% to 49.4%, stress in 12.6% to 50.6%, and depression in 15.9% to 28.9%. ^14^ We did not find an association between insomnia and sex in the present study; however, women expressed higher insomnia in previous studies. ^40^^,^^43^^–^^45^

We found a significant association between insomnia and symptoms of stress, depression, and anxiety by univariate analysis. However, on logistic regression analysis, we observed a significant independent relationship between stress and sleeplessness (p-value < 0.001). At the same time, there was no discernible correlation between anxiety, sadness, sex, education school boards, or type of educational institution. Association of insomnia symptoms with depression, ^37^^,^^40^^,^^41^^,^^45^^,^^46^ anxiety, ^15^^,^^37^^,^^40^^,^^41^^,^^45^^,^^46^ and stress in various populations were present in earlier research. ^45^^–^^47^ Multiple other factors in different people, such as older age, being an HCP, education level, and living in a city or near the epicenter ^15^^,^^16^^,^^37^^,^^40^^,^^43^^,^^45^ were significantly associated with insomnia.

The study had a few limitations. The sample size was relatively small, which could impact the generalizability of the results. Additionally, there was an underrepresentation of male teachers, which may have skewed the findings toward a female perspective. Lastly, there is a possibility of bias in the responses to the questionnaire, which could have influenced the results. Further, though there was a significant association between insomnia and psychological symptoms, it is unclear if insomnia causes psychological symptoms or vice versa because the study was cross-sectional. We did not establish the causality of insomnia and psychological symptoms. We speculated it to be a blend of adopting COVID-appropriate behaviors at school, worry of being infected, and school reopening after a halt.

The study's strengths included using a standard questionnaire to measure teachers' levels of insomnia prevalence (by ISI) and manifestations of stress, anxiety, and depression (by DASS 21). In addition, this study is the first one in the Indian context to assess insomnia and psychological symptoms in teachers who joined back to work at school after a hiatus.

Insomnia will impact the performance of teachers by affecting concentration, memory, and decision-making abilities. Strategic interventions such as cognitive behavioral therapy, mindfulness practices, ^48^ and various sleep hygiene techniques will improve sleep quality and reduce stress, anxiety, and depressive symptoms in the long run.

It will be prudent if a longitudinal study is conducted in the future to establish the causality of insomnia and psychological symptoms and explore the effectiveness of different interventions to reduce insomnia among teachers.

## Conclusions

To conclude, we reported insomnia in 37.9 % of teachers after resuming face-to-face classes in schools during the COVID-19 pandemic. Symptoms of stress, depression, and anxiety were present in 20.2%, 30.6%, and 45.2% of teachers, respectively. An independent risk factor for insomnia was presence of psychological stress. Identifying sleeplessness and psychological issues in teachers and addressing them as early as possible is essential for an effective teaching atmosphere at school and overall well-being.

## Data Availability

Open Scientific Framework **:** Insomnia in teachers with the resumption of in-person instruction at schools amidst the COVID-19 pandemic–a cross-sectional study. **2023**. https://osf.io/b4c3u. ^31^ This dataset contains the following underlying data:
• Excel Data file • Data code Key Open Scientific Framework: Insomnia in teachers with the resumption of in-person instruction at schools amidst the COVID-19 pandemic–a cross-sectional study. **2023**. https://osf.io/b4c3u. ^31^ This dataset contains the following underlying extended data:
• Parent information sheet • Informed consent form • Study Questionnaire Open Scientific Framework: STROBE checklist for Insomnia in teachers with the resumption of in-person instruction at schools amidst the COVID-19 pandemic–a cross-sectional study. **2023**. https://osf.io/b4c3u Data are available under the terms of the Creative Commons Attribution 4.0 International license (CC-BY 4.0).
