# Supplementary material for: Insomnia in teachers at the time of resumption of in-person instruction at schools amidst the COVID-19 pandemic: A cross-sectional study
Source: F1000Res. 2024 Sep 5;12:1217. Originally published 2023 Sep 26. [Version 2] doi: 10.12688/f1000research.141274.2 (PMC11425037; doi:10.12688/f1000research.141274.2)
Supplement: Peer review history

# Reviewer report: Reviewer response for version 2

**DOI:** 10.5256/f1000research.171018.r320976

**Published:** 2024-09-25

**Authors:** Sreejayan Kongasseri

**Affiliations:** 1 Psychiatrist, Northern Health, Melbourne, Australia

No further comments to make

 The authors have responded appropriately

Is the work clearly and accurately presented and does it cite the current literature?

Yes

If applicable, is the statistical analysis and its interpretation appropriate?

Yes

Are all the source data underlying the results available to ensure full reproducibility?

Yes

Is the study design appropriate and is the work technically sound?

Yes

Are the conclusions drawn adequately supported by the results?

Partly

Are sufficient details of methods and analysis provided to allow replication by others?

Yes

Reviewer Expertise:

Psychiatry, mental health, addictions

I confirm that I have read this submission and believe that I have an appropriate level of expertise to confirm that it is of an acceptable scientific standard.

# Reviewer report: Reviewer response for version 1

**DOI:** 10.5256/f1000research.154704.r226966

**Published:** 2024-01-04

**Authors:** Ravichandra Karkal

**Affiliations:** 1 Department of Psychiatry, Yenepoya Medical College Hospital, Mangaluru, Karnataka, India

The following modifications are required for the manuscript.

 The background section of abstract is lengthy and can be summarised as follows- The COVID-19 pandemic has affected the sleep and mental health of many people, including teachers who had to adapt to online teaching and then resume in-person instruction at schools. This study aimed to estimate the prevalence of insomnia and its association with stress, anxiety, and depression among teachers in India after schools reopened following a hiatus due to the pandemic.

 The introduction is too lengthy and contains some irrelevant information **.** For example, the paragraph on the effects of insomnia on health could be omitted or moved to the discussion section, as it is not directly related to the research question.

 Replace Ref no. 5 with following as the current reference is from teachers from special education school and may not be applicable to all teachers in regular schools. Agyapong, B., Obuobi-Donkor, G., Burback, L., & Wei, Y. (2022). Stress, Burnout, Anxiety and Depression among Teachers: A Scoping Review. *International Journal of Environmental Research and Public Health*, 19. (Ref. 1). 

 In methodology: The details of the tools (ISI and DASS 21) can be made concise and psychometric properties can be highlighted rather than describing the tool in great details.

 In results section- "The presence of anxiety or depressive symptoms, sex, school educational boards, or the type of institution did not significantly correlate with insomnia in teachers."-- Use the term “association” instead of “correlation” to describe the relationship between insomnia and psychological symptoms, as this is more appropriate for categorical variables. 

 Table 4 title - Logistic regression

 Table 4- Use "p" instead of "p value"

 Those teachers who were stressed out had a 6.4 times higher incidence of insomnia (odds ratio).- Replace incidence with odds. Those teachers who were stressed out had a 6.4 times higher odds of reporting insomnia.

 Last paragraph of results can be replaced with following- On univariate analysis using Chi-square tests, we found a significant association of insomnia with symptoms of stress, depression, and anxiety (p < 0.001 for all). On multivariate analysis using logistic regression, we found that only stress was an independent factor for insomnia (95% CI: 1.5-28.3, P = 0.01). Those teachers who were stressed had a 6.4-fold higher odds of insomnia than those who were not stressed. The presence of anxiety or depressive symptoms, sex, school educational boards, and type of institution were neither independently nor jointly associated with insomnia.

 In discussion 6th paragraph- Avoid using “by” to introduce the statistical test, as this can be ambiguous and misleading. Instead, use “using” or “with” to indicate the method of analysis.

 In discussion section focus on the comparison of the prevalence and severity of insomnia and psychological symptoms among teachers with other populations, such as health care workers, general public, and COVID-19 patients. The authors could use more specific terms to describe the differences, such as higher, lower, similar, or comparable.

 Discuss the possible mechanisms and explanations for the association between insomnia and stress, as well as the lack of association between insomnia and other variables, such as anxiety, depression, sex, school boards, and type of institution. The authors could provide (wherever feasible)more evidence and reasoning to support their hypotheses, such as biological, psychological, or social factors that could mediate or moderate the relationship between insomnia and stress.

Is the work clearly and accurately presented and does it cite the current literature?

Yes

If applicable, is the statistical analysis and its interpretation appropriate?

Yes

Are all the source data underlying the results available to ensure full reproducibility?

Yes

Is the study design appropriate and is the work technically sound?

Yes

Are the conclusions drawn adequately supported by the results?

Yes

Are sufficient details of methods and analysis provided to allow replication by others?

Yes

Reviewer Expertise:

Mood disorders, Social media addiction, Insomnia

I confirm that I have read this submission and believe that I have an appropriate level of expertise to confirm that it is of an acceptable scientific standard.

## References

[1] : Stress, Burnout, Anxiety and Depression among Teachers: A Scoping Review. *Int J Environ Res Public Health* .2022;19(17) : 10.3390/ijerph191710706 10.3390/ijerph191710706 36078422 PMC9518388

# Response

**Published:** 2024-08-21

**Authors:** Sowmini Padmanabh Kamath

**Affiliations:** Kasturba Medical College, Mangalore, India

The following modifications are required for the manuscript.

 1. The background section of abstract is lengthy and can be summarised as follows- The COVID-19 pandemic has affected the sleep and mental health of many people, including teachers who had to adapt to online teaching and then resume in-person instruction at schools. This study aimed to estimate the prevalence of insomnia and its association with stress, anxiety, and depression among teachers in India after schools reopened following a hiatus due to the pandemic.

**Author Response: **As suggested, changes have been made in the background section of the abstract.

 2. The introduction is too lengthy and contains some irrelevant information **.** For example, the paragraph on the effects of insomnia on health could be omitted or moved to the discussion section, as it is not directly related to the research question.

**Author Response: ** As suggested, the changes have been made. The paragraph on the effects of insomnia on health has been omitted in the introduction section. 

 3. Replace Ref no. 5 with following as the current reference is from teachers from special education school and may not be applicable to all teachers in regular schools. Agyapong, B., Obuobi-Donkor, G., Burback, L., & Wei, Y. (2022). Stress, Burnout, Anxiety and Depression among Teachers: A Scoping Review. *International Journal of Environmental Research and Public Health*, 19. (Ref. 1). 

**Author Response: ** The reference 5  has been updated as suggested

 Agyapong B, Obuobi-Donkor G, Burback L, Wei Y. Stress, Burnout, Anxiety and Depression among Teachers: A Scoping Review. Int J Environ Res Public Health. 2022 Aug 27;19(17):10706. https://doi.org/10.3390/ijerph191710706

 4. In methodology: The details of the tools (ISI and DASS 21) can be made concise and psychometric properties can be highlighted rather than describing the tool in great details.

**Author Response: ** The details of the tools have been concised, and psychometric properties are highlighted with relevant references. 

 5.In results section- "The presence of anxiety or depressive symptoms, sex, school educational boards, or the type of institution did not significantly correlate with insomnia in teachers."-- Use the term “association” instead of “correlation” to describe the relationship between insomnia and psychological symptoms, as this is more appropriate for categorical variables.

**Author Response: ** As suggested, the term association is used instead of correlation

 6. Table 4 title - Logistic regression

 Table 4- Use "p" instead of "p value"

**Author Response: ** The necessary changes are done in Table 4.

 7. Those teachers who were stressed out had a 6.4 times higher incidence of insomnia (odds ratio).- Replace incidence with odds. Those teachers who were stressed out had a 6.4 times higher odds of reporting insomnia.

**Author Response: ** The word incidence has been replaced by the word odds - Those teachers who were stressed had 6.4 times higher odds of reporting insomnia.

 8. Last paragraph of results can be replaced with following- On univariate analysis using Chi-square tests, we found a significant association of insomnia with symptoms of stress, depression, and anxiety (p < 0.001 for all). On multivariate analysis using logistic regression, we found that only stress was an independent factor for insomnia (95% CI: 1.5-28.3, P = 0.01). Those teachers who were stressed had a 6.4-fold higher odds of insomnia than those who were not stressed. The presence of anxiety or depressive symptoms, sex, school educational boards, and type of institution were neither independently nor jointly associated with insomnia.

**Author Response: ** As suggested, last paragraph of results has been reframed.

 9. In discussion 6th paragraph- Avoid using “by” to introduce the statistical test, as this can be ambiguous and misleading. Instead, use “using” or “with” to indicate the method of analysis.

**Author Response: ** Appropriate suggested word has been used to indicate the method of analysis

 10. In discussion section focus on the comparison of the prevalence and severity of insomnia and psychological symptoms among teachers with other populations, such as health care workers, general public, and COVID-19 patients. The authors could use more specific terms to describe the differences, such as higher, lower, similar, or comparable.

**Author Response: ** The prevalence of insomnia and psychological symptoms among teachers with other populations such as health care workers, the general public, and COVID-19 patients have been discussed with specific terms such as higher, lower, similar, and comparable to describe differences. Relevant references added

 11. Discuss the possible mechanisms and explanations for the association between insomnia and stress, as well as the lack of association between insomnia and other variables, such as anxiety, depression, sex, school boards, and type of institution. The authors could provide (wherever feasible)more evidence and reasoning to support their hypotheses, such as biological, psychological, or social factors that could mediate or moderate the relationship between insomnia and stress.

**Author Response: ** The explanation has been described in the last few paragraphs (before the limitations section) of the discussion, with relevant references added

# Reviewer report: Reviewer response for version 1

**DOI:** 10.5256/f1000research.154704.r226960

**Published:** 2023-12-20

**Authors:** Sreejayan Kongasseri

**Affiliations:** 1 Psychiatrist, Northern Health, Melbourne, Australia

This is a well conducted study with a strong methodology. Authors have used standardised scales and have explained the sampling method elaborately. Appropriate statistical methods have been used and results are well presented.

 Here are few suggestions:

 This is a cross sectional study which means that the prevalence of insomnia has been estimated at a certain point of time ( when the  in person teaching resumed). However authors have used words in ways as if the in person teaching was causal to the insomnia and stress.

 Title “ Insomnia in teachers with the resumption of in-person” suggests causality. “ at the time of” will be more appropriate here

 “ We observed that 37.9 % of teachers expressed the presence of insomnia (subthreshold - 25 % and clinical -12.9 %) after resuming face-to-face classes at school amid the COVID-19 pandemic” instead of after “ at the time of”.

 “ Similarly, a review study documented that the COVID-19 pandemic is linked with a significant increase in subthreshold insomnia but not with moderate or severe symptoms of insomnia.34” this sentence can come later with a statement that it was not possible to know if the prevalence had increased.

 In the flow diagram the reasons for exclusions could have been mentioned. Medical condition has been reported as reason for exclusion. It is difficult to imagine a condition with which a teacher can attend work but get excluded from the study. If the authors excluded them due to conditions that caused insomnia then it would be clearer if those excluded conditions were mentioned. Were prior psychiatric conditions excluded? “16 teachers did not have internet facility” is not clear. The survey was sent electronically. If they did not have internet access they would not have known about the study.

 Some more points to include in discussion ( not covered)

 More women than men

 Lower prevalence in this study when compared to existing literature. Even though India had a high mortality rate during COVID.

 “Lastly, there is a possibility of bias in the responses to the questionnaire” this statement is not clear. Needs some elaboration.

Is the work clearly and accurately presented and does it cite the current literature?

Yes

If applicable, is the statistical analysis and its interpretation appropriate?

Yes

Are all the source data underlying the results available to ensure full reproducibility?

Yes

Is the study design appropriate and is the work technically sound?

Yes

Are the conclusions drawn adequately supported by the results?

Partly

Are sufficient details of methods and analysis provided to allow replication by others?

Yes

Reviewer Expertise:

Psychiatry, mental health, addictions

I confirm that I have read this submission and believe that I have an appropriate level of expertise to confirm that it is of an acceptable scientific standard, however I have significant reservations, as outlined above.

# Response

**Published:** 2023-12-26

**Authors:** Sowmini Padmanabh Kamath

**Affiliations:** Kasturba Medical College, Mangalore, India

**This is a cross sectional study which means that the prevalence of insomnia has been estimated at a certain point of time ( when the  in person teaching resumed). However authors have used words in ways as if the in person teaching was causal to the insomnia and stress.Title “ Insomnia in teachers with the resumption of in-person” suggests causality. “ at the time of” will be more appropriate here**

 A. We have changed the title as follows: Insomnia in teachers at the time of resumption of in-person instruction at schools amidst the COVID-19 pandemic: A cross-sectional study.

**“ We observed that 37.9 % of teachers expressed the presence of insomnia (subthreshold - 25 % and clinical -12.9 %) after resuming face-to-face classes at school amid the COVID-19 pandemic” instead of after “ at the time of”.**

 A. We have changed the sentence as follows:We observed that 37.9 % of teachers expressed the presence of insomnia (subthreshold - 25 % and clinical -12.9 %) at the time of resuming face-to-face classes at school amid the COVID-19 pandemic

**“ Similarly, a review study documented that the COVID-19 pandemic is linked with a significant increase in subthreshold insomnia but not with moderate or severe symptoms of insomnia.34” this sentence can come later with a statement that it was not possible to know if the prevalence had increased.**

 A.  As suggested, the sentence has been placed in the subsequent paragraphs- Similarly, a review study has documented that the COVID-19 pandemic is linked with a significant increase in subthreshold insomnia but not with moderate or severe symptoms of insomnia.34” and it was not possible to know if the prevalence had increased in our study.

**In the flow diagram the reasons for exclusions could have been mentioned. Medical condition has been reported as reason for exclusion. It is difficult to imagine a condition with which a teacher can attend work but get excluded from the study. If the authors excluded them due to conditions that caused insomnia then it would be clearer if those excluded conditions were mentioned. Were prior psychiatric conditions excluded? “16 teachers did not have internet facility” is not clear. The survey was sent electronically. If they did not have internet access they would not have known about the study.**

 A. About 56 teachers were excluded from the study. A total of 40 teachers did not consent to the study because of unwillingness in 28 and medical conditions that affect sleep in 12 {diabetes in 7, heart disease in 3, and asthma in 2}. Once the principal sent the questionnaire link to the WhatsApp group, those teachers with the above conditions expressed dissent.

 The subjects approached did not mention any presence of prior psychiatric conditions, probably due to fear of social stigma. We could not probe further on the same because of feasibility issues.

 We have now clarified that 16 teachers did opt out of the study since they mentioned they have poor internet connectivity issues since they resided in remote rural areas and came to work at school in the city.

 The explanation is done in the flow diagram and enclosed. 

**Some more points to include in the discussion (not covered)**

**More women than men**

 A. It is well known that females make up most teachers in a large number of schools. Likewise, our selected schools were populated with female teachers. Thus, our study also had more participation from female teachers (87.1%). We did not find an association between insomnia and sex in the present study; however, women expressed higher insomnia in previous studies. ^40^^ ,^^43^^ –^^45^

**There is a lower prevalence in this study when compared to existing literature. Even though India had a high mortality rate during COVID.**

 A. Earlier studies ^15,16,35,37-42^ had insomnia prevalence ranging between 13.32 % and 57.2 % across various populations in various countries, with data collection done during the peak COVID-19 pandemic times when a lot of unprecedented and uncertain changes were occurring in people's daily lives.

 We conducted our study after a prolonged hiatus of lockdown. In addition, improved knowledge and treatment facilities for COVID-19 infection and adapting to COVID-appropriate behaviors along with effective vaccination drive by the Indian government must have contributed to the lesser prevalence compared to a few earlier studies. The reopening of schools after a hiatus, the difficulties in implementing and adopting new routines, and the possibility of an infection's unpredictable reappearance were the combined reasons for insomnia and the psychological symptoms, which we could not prove to be causal.

 “ **Lastly, there is a possibility of bias in the responses to the questionnaire.” This statement is not clear. Needs some elaboration**

 A. We have removed the sentence since both questionnaire scales have demonstrated reliability and validity in populations.

# Response

**Published:** 2024-08-21

**Authors:** Sowmini Padmanabh Kamath

**Affiliations:** Kasturba Medical College, Mangalore, India

This is a well-conducted study with a strong methodology. Authors have used standardised scales and have explained the sampling method elaborately. Appropriate statistical methods have been used and results are well presented.

 Here are a few suggestions:

 1. This is a cross sectional study which means that the prevalence of insomnia has been estimated at a certain point of time ( when the  in person teaching resumed). However authors have used words in ways as if the in person teaching was causal to the insomnia and stress.

 Title “ Insomnia in teachers with the resumption of in-person” suggests causality. “ at the time of” will be more appropriate here.

**Author Response:** We have changed the title as follows: “Insomnia in teachers at the time of resumption of in-person instruction at schools amidst the COVID-19 pandemic: A cross-sectional study”.

 2. “ We observed that 37.9 % of teachers expressed the presence of insomnia (subthreshold - 25 % and clinical -12.9 %) after resuming face-to-face classes at school amid the COVID-19 pandemic” instead of after “ at the time of”.

**Author Response:** We have changed the sentence as follows: we observed that 37.9% of teachers expressed the presence of insomnia (subthrhold-25% and clinical -12.9%) at the time of resuming face -to face classes at school amid the COVID -19 pandemic.

 3. “ Similarly, a review study documented that the COVID-19 pandemic is linked with a significant increase in subthreshold insomnia but not with moderate or severe symptoms of insomnia.34” this sentence can come later with a statement that it was not possible to know if the prevalence had increased.

**Author Response:** As suggested, the sentence has been placed in the subsequent paragraphs- 

 A review study has documented that the COVID-19 pandemic is linked with a significant increase in subthreshold insomnia but not with moderate or severe symptoms of insomnia, ^42  ^and it was not possible to know if the prevalence had increased in our study. The reference number has been updated. 

 4. In the flow diagram the reasons for exclusions could have been mentioned. Medical condition has been reported as reason for exclusion. It is difficult to imagine a condition with which a teacher can attend work but get excluded from the study. If the authors excluded them due to conditions that caused insomnia then it would be clearer if those excluded conditions were mentioned. Were prior psychiatric conditions excluded? “16 teachers did not have internet facility” is not clear. The survey was sent electronically. If they did not have internet access, they would not have known about the study.

**Author Response:** About 56 teachers were excluded from the study. A total of 40 teachers did not consent to the study because of unwillingness in 28 and medical conditions that affect sleep in 12 {diabetes in 7, heart disease in 3, and asthma in 2}. Once the principal sent the questionnaire link to the What’s app group, those teachers with the above conditions expressed dissent. About 16 teachers could open the questionnaire link; however, due to internet issues and technical glitches, they could not either complete or submit the questionnaire and, therefore, decided to opt out of the study.

 The subjects approached did not mention any presence of prior psychiatric conditions, probably due to fear of social stigma. We could not probe further into this because of feasibility issues.

 The explanation is done in the flow diagram and enclosed.

 Some more points to include in the discussion ( not covered)

 5. More women than men

**Author Response:** It is well-known that females make up most teachers in many schools. Likewise, our selected schools have female teachers, contributing to 87.1% of participation. Higher psychological symptoms in teachers have been reported earlier, ^24,71,^ and in the current study, we found a significant association between symptoms of anxiety and sex. ^25 ^However, we did not find an association between insomnia and sex in the present study, although women expressed higher insomnia in previous studies. ^52,^ ^59,72,73^

 6. Lower prevalence in this study when compared to existing literature. Even though India had a high mortality rate during COVID.

**Author Response:** There were variations in the insomnia prevalences in previous studies across a spectrum of populations ^43,44,45,53,54,56,58^ in different countries, with data collection done during other times of the COVID-19 pandemic, with some conducted at its peak times when many unprecedented and uncertain changes were occurring in people's daily lives. We conducted our study after a prolonged hiatus of lockdown. In addition, improved knowledge and treatment facilities for COVID-19 infection and adapting to COVID-19 appropriate behaviors along with an effective vaccination drive by the Indian government might have contributed to the lower prevalence compared to a few earlier studies. The reopening of schools after a hiatus, the difficulties in implementing and adopting new routines, and the possibility of an infection’s unpredictable reappearance were the combined reasons for insomnia and the psychological symptoms, which we could not prove to be causal.

 7. “Lastly, there is a possibility of bias in the responses to the questionnaire” this statement is not clear. Needs some elaboration.

**Author Response:** We have removed the sentence since both questionnaire scales have demonstrated reliability and validity in populations.
